# Diversity of mitophagy pathways at a glance

**DOI:** 10.1242/jcs.259748

**Published:** 2022-12-12

**Authors:** Ian G. Ganley, Anne Simonsen

**Affiliations:** ^1^ MRC Protein Phosphorylation and Ubiquitylation Unit, School of Life Sciences, University of Dundee, Dundee DD1 5EH, UK; ^2^ Department of Molecular Medicine, Institute of Basic Medical Sciences, University of Oslo, 0372 Oslo, Norway; ^3^ Centre for Cancer Cell Reprogramming, Institute of Clinical Medicine, University of Oslo, 0318 Oslo, Norway; ^4^ Department of Molecular Cell Biology, Institute for Cancer Research, Oslo University Hospital Montebello, 0379 Oslo, Norway

**Keywords:** Mitochondria, Mitophagy, SLR, PINK1, Parkin, HIF1, BNIP3, NIX, Selective autophagy

## Abstract

Mitochondria are crucial organelles that play a central role in various cell signaling and metabolic pathways. A healthy mitochondrial population is maintained through a series of quality control pathways and requires a fine-tuned balance between mitochondrial biogenesis and degradation. Defective targeting of dysfunctional mitochondria to lysosomes through mitophagy has been linked to several diseases, but the underlying mechanisms and the relative importance of distinct mitophagy pathways *in vivo* are largely unknown. In this Cell Science at a Glance and the accompanying poster, we describe our current understanding of how parts of, or whole, mitochondria are recognized by the autophagic machinery and targeted to lysosomes for degradation. We also discuss how this might be regulated under different physiological conditions to maintain mitochondrial and cellular health.

## Teaser

**Summary:** This Cell Science at a Glance provides insights into how mitochondria are recognized and targeted to lysosomes for degradation under different physiological conditions to maintain cellular homeostasis.

## Introduction

**Figure:**
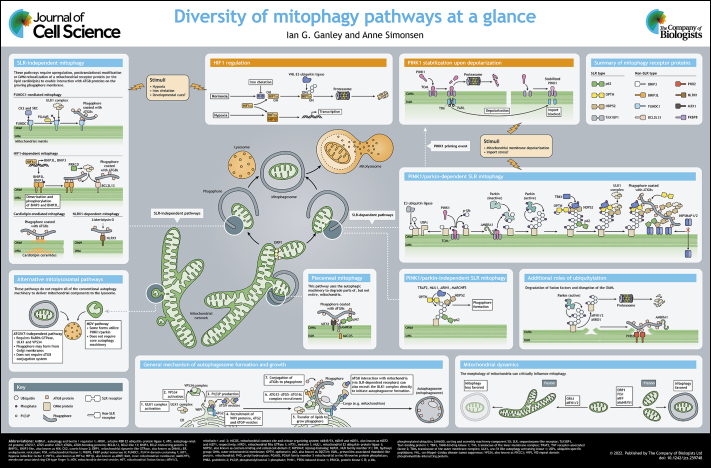


Mitophagy is a type of selective autophagy that involves sequestration of mitochondria by a double-membrane phagophore that closes to form a mitophagosome, which upon fusion with a lysosome leads to degradation of the sequestered mitochondrial material. Mitophagosome biogenesis can be initiated by various cellular and environmental stressors, such as starvation, hypoxia and mitochondrial damage, but mitophagy is also important for the regulation of mitochondrial abundance in response to oocyte fertilization, erythroid cell maturation, stem cell pluripotency and neuronal differentiation (Montava-Garriga and Ganley, 2020; Ng et al., 2021; Onishi et al., 2021). Importantly, mitochondrial stress is a hallmark of several diseases, such as neurodegeneration and cancer (Bernardini et al., 2017; Moehlman and Youle, 2020), but further elucidation of different mitolysosomal pathways is needed to specifically address whether lysosomal degradation of mitochondria prevents or rather promotes disease development.

Several mitochondrial proteins and lipids have been implicated in the recruitment and activation of the core autophagy machinery (see Box 1) to initiate mitophagosome biogenesis. Mitophagy generally involves the binding of mitophagy receptor proteins to ATG8-homolog proteins (hereafter referred to as ATG8 proteins or ATG8s) in the mitophagosome membrane. Such mitophagy receptors can either be integral mitochondrial proteins localized to the outer mitochondrial membrane (OMM) or soluble autophagy receptor proteins of the sequestosome-like receptor (SLR) family that bind to ubiquitylated OMM proteins. The complexities of the molecular machineries involved in different mitophagy pathways, which are induced in response to various cellular and environmental stressors, are just starting to be unveiled, and little is known about how these machineries are regulated in time and space *in vivo*. Here, we provide a general overview of our current knowledge about the signals and mechanisms involved in different mitophagy pathways, which are summarized in the accompanying poster.

**Box 1. Core autophagy machinery**

Macroautophagy, and hence mitophagy, requires proteins encoded by the core autophagy-related genes (*ATGs*) that are essential for autophagosome formation (for an in-depth review see Bento et al., 2016; Chang et al., 2021). These ATG proteins operate in complexes that can be divided into four main functional systems. (1) The ULK1 kinase complex, consisting of the ULK1 serine/threonine kinase, ATG13, ATG101 and FIP200, is thought to be the most upstream complex and acts as a node to convert stress signals into autophagosome formation. Major autophagy-regulating signaling pathways, such as the mechanistic target of rapamycin (MTOR) or AMP-activated protein kinase (AMPK) pathways, all converge at this kinase complex. (2) A major downstream target of the ULK1 complex is the next system, the class III phosphatidylinositol 3-kinase complex I, which consists of the VPS34 (PIK3C3) catalytic subunit, VPS15, Beclin1 and ATG14. This complex phosphorylates phosphatidylinositol at position 3 of its inositol ring to produce phosphatidylinositol 3-phosphate [PI(3)P]. This occurs on a specialized endoplasmic reticulum subdomain, termed the omegasome, which is thought to act as a cradle or platform to help form the autophagosome. Here, PI(3)P recruits downstream PI(3)P-binding proteins, including the WIPI family of proteins, that aid in the targeting of the next autophagy core system: the ubiquitin-like conjugation system. (3) The ubiquitin-like conjugation system conjugates the ubiquitin-like ATG8 family of proteins (see Box 2) to phosphatidylethanolamine (PE) in the growing autophagosomal membrane. This is achieved by two series of ubiquitin-like conjugation reactions. In the first, the ubiquitin-like protein ATG12 is conjugated to ATG5 via ATG7 (the E1 enzyme) and ATG10 (an E2 enzyme). The ATG12–ATG5 conjugate then binds to ATG16L1 to form the ATG12–ATG5–ATG16L1 complex, which acts as a novel E3 ligase enzyme in the conjugation of activated ATG8 proteins to PE. Prior to their lipidation, ATG8s are cleaved by ATG4 and conjugated to ATG7 and ATG3 (an E2 enzyme). The correct membrane targeting of ATG8 conjugation is achieved by the ATG12–ATG5–ATG16L1 complex through its direct membrane binding and interaction with the PI(3)P-binding protein WIPI2. As discussed in Box 2, the ATG8 proteins play key roles in controlling autophagosome size, cargo selection, fusion and lysosomal degradation. (4) The final system involved in autophagosome formation is the ATG9 system. ATG9A and ATG9B, the only transmembrane ATG proteins, are lipid scramblases that together with the lipid transfer proteins ATG2A and ATG2B play a key role in transferring lipid to the growing autophagosome.

**Box 2. ATG8 proteins and LIR motifs**

Mammalian ATG8 proteins include seven paralogs of the single yeast Atg8 protein and can be grouped in two subfamilies: microtubule-associated protein light chain 3 proteins (referred to collectively as LC3 and comprising LC3A, encoded by *MAP1LC3A*; LC3B, encoded by *MAP1LC3B*; LC3B2, encoded by *MAP1LC3B2*; and LC3C, encoded by *MAP1LC3C*) and γ-aminobutyric acid receptor-associated proteins (GABARAP, GABARAPL1, GABARAPL2). ATG8s are ubiquitin-like proteins that become covalently linked to PE on both sides of the forming autophagosome double membrane (see Box 1). The exact functions of the various ATG8s are still poorly understood, but they do have in common the ability to bind to proteins containing specific LIR motifs (for an in-depth review please see Johansen and Lamark, 2020; Nguyen and Lazarou, 2022; Slobodkin and Elazar, 2013; Stolz et al., 2014). The core LIR motif consists of an aromatic amino acid and a hydrophobic amino acid that are separated by any two amino acids – [W/F/Y]-X_1_-X_2_-[I/L/V] – and is often surrounded by negatively charged residues or phosphorylatable serine/threonine residues that can regulate interactions between the LIR and ATG8 proteins. The side chains of the core aromatic and hydrophobic LIR residues interact with two hydrophobic pockets on the surface of ATG8s. While some LIR-containing proteins appear to interact with all ATG8s, others might have a preference for LC3 or GABARAP family members. Based on a comparison of LIR sequences and mutation analysis, a specific GABARAP-interacting motif (GIM) has been defined: [W/F]-[V/I]-X_2_-V. The main function of ATG8s appears to be to recruit LIR-containing proteins that act as autophagy receptors or autophagy adaptors. Autophagy receptors link the cargo to be degraded to ATG8s on the inner autophagic membrane, leading to lysosomal delivery and degradation of both cargo and receptor proteins. In contrast, autophagy adaptor proteins interact with ATG8s on the outer autophagic membrane to facilitate autophagosome formation, autophagosome transport or autophagosome–lysosome fusion, without being themselves degraded by autophagy. It is, however, important to note that ATG8s are not absolutely required for autophagosome biogenesis, but rather that they appear to regulate autophagosome size and kinetics. Moreover, conjugation of ATG8s to single membranes (referred to as CASM) can take place in processes other than autophagy, such as secretion, phagocytosis and endosomal repair.

## Key mitophagy stimuli

For mitophagy receptors to trigger mitophagy, they must first be recruited to and/or activated at the target mitochondrion. While this is likely to occur under many diverse pathophysiological scenarios, our understanding of the nature of stimuli that lead to this is limited. However, mitochondrial depolarization and hypoxia-inducible factor 1 (HIF1)-dependent signaling play important roles in many of the mitophagy pathways studied to date.

### Mitochondrial depolarization

Normal mitochondrial function requires polarization across the inner mitochondrial membrane (IMM). This proton electrochemical gradient, which is generated by the electron transport chain, is essential for ATP production and other mitochondrial transport functions. It is a key indicator of mitochondrial health, and depolarization frequently occurs as a result of mitochondrial damage or dysfunction. Depolarization can also act as a critical early step in the triggering of mitophagy, to maintain mitochondrial homeostasis (Ng et al., 2021). A major initial consequence of mitochondrial depolarization is an impairment or block of mitochondrial protein import (Zorova et al., 2018). This leads to stabilization of PTEN-induced kinase 1 (PINK1) in the translocase of the outer membrane complex (TOM complex) (Lazarou et al., 2012; Okatsu et al., 2013) and, as detailed below, mitophagy initiation. Indeed, inhibition of mitochondrial import itself is believed to be a critical mitophagy signaling event, and blockage of this by import stress, for example, is sufficient to trigger mitophagy (Burbulla et al., 2014; Jin and Youle, 2013). Depolarization also leads to relocalization, or accessibility to the mitophagy machinery, of other key mitochondrial components, such as the nipsnap homologs NIPSNAP1 and NIPSNAP2 (Princely Abudu et al., 2019), prohibitin 2 (PHB2) (Wei et al., 2017) and the mitochondrial lipid cardiolipin (Chu et al., 2013), all of which help to establish a combinatorial set of interactions that help to mount a robust mitophagy response to mitochondrial damage. Experimentally, depolarization-induced mitophagy is often stimulated using small-molecule mitochondrial uncouplers, such as carbonyl cyanide 3-chlorophenylhydrazone (CCCP) (Narendra et al., 2008), or a combination of oligomycin A and antimycin A1 (Allen et al., 2013).

### HIF1α stabilization

#### Hypoxia

Mitochondria are the major oxygen-consuming organelles of the cell; hence, they must be able to adapt to changes in oxygen tension and demand. A failure to do this under low (or high) oxygen tension can lead to increased reactive oxygen species (ROS) production, mitochondrial damage and cell death. Under these conditions, in particular in hypoxia, mitophagy can be activated. An important response to low oxygen is stabilization of HIF1α (also known as HIF1A), which drives a transcriptional program to optimize cell survival under low oxygen, including the upregulation of SLR-independent mitophagy via transcription of the OMM mitophagy receptors BCL2-interacting protein 3 (BNIP3) and BNIP3-like (BNIP3L, also known as NIX) (Bellot et al., 2009; Zhao et al., 2020) (see poster). However, hypoxia can also induce mitophagy independently of HIF1; for example, the mitophagy receptor FUN14 domain-containing 1 (FUNDC1) can activate mitophagy under hypoxia in platelets, which lack a nucleus and, hence, transcription (Zhang et al., 2016).

#### Iron depletion

In addition to low oxygen, HIF1α can be stabilized by other factors, such as intracellular iron depletion or a reduction in the levels of the tricarboxylic acid cycle metabolite α-ketoglutarate. Both iron and α-ketoglutarate are essential co-factors for the family of prolyl hydroxylases that are responsible for the targeted degradation of HIF1α; hence, conditions that drastically reduce these components can lead to HIF1-dependent mitophagy under normoxic conditions (Pugh, 2016). While this can occur physiologically through changes in metabolism, it can also be induced pharmacologically with the use of iron chelators, such as deferiprone (DFP) or desferrioxamine (DFO), and synthetic α-ketoglutarate analogs including dimethyloxalylglycine (DMOG) (Allen et al., 2013).

#### Differentiation and metabolism

HIF1α stabilization also results in cellular metabolic remodeling through an upregulation of genes encoding glycolytic enzymes (Kierans and Taylor, 2021). Enhancement of glycolysis affords conditions permissive for mitophagy: it helps to maintain energy balance by providing increased non-mitochondrial ATP production. However, it also appears that mitophagy can facilitate this glycolytic shift, as BNIP3L-mediated mitophagy is critical for the glycolytic change that drives differentiation during neuronal development and macrophage activation (Esteban-Martinez et al., 2017).

## Different mitophagy pathways

Our mechanistic knowledge of mitophagy in response to different mitochondrial stressors is still limited, making it difficult to segregate distinct pathways. With our current understanding, two main types of mitophagy can be distinguished based on the requirement for soluble mitophagy receptor proteins of the SLR family versus mitophagy receptors being localized to the OMM (see poster). The former is generally dependent on ubiquitylation of OMM proteins by the RING-between-RING (RBR) ubiquitin–protein ligase parkin (encoded by *PRKN*) and PINK1, both of which are mutated in some forms of hereditary Parkinson's disease (McWilliams and Muqit, 2017). Given this, this type of mitophagy is often referred to as PINK1/parkin-dependent mitophagy. However, there are now known examples of SLR-dependent pathways that appear to be independent of PINK1 and parkin, and in the following sections, we therefore discuss these two pathways separately and in contrast to the SLR-independent mitophagy pathways that rely on mitochondrial receptors.

### SLR-dependent mitophagy – a soluble receptor pathway

#### PINK1/parkin-dependent SLR mitophagy

This ubiquitin-dependent mitophagy pathway is typically stimulated by mitochondrial membrane depolarization, leading to stabilization of the serine/threonine kinase PINK1 on the OMM (see poster). PINK1 is normally imported into healthy mitochondria, leading to its cleavage by mitochondrial proteases (Greene et al., 2012; Jin et al., 2010) and subsequent proteasomal degradation (Yamano and Youle, 2013). However, when at the OMM, PINK1 becomes activated, leading to phosphorylation of ubiquitin (at Ser65) conjugated to OMM proteins, and mitochondrial recruitment and activation of the E3 ubiquitin ligase parkin (Kane et al., 2014; Kazlauskaite et al., 2014; Okatsu et al., 2015, 2013; Wauer et al., 2015). Parkin further ubiquitylates OMM proteins to generate polyubiquitin chains (Narendra et al., 2008). Other E3 ubiquitin ligases have been found to cooperate with parkin in polyubiquitylation of OMM proteins and mitophagy, including mitochondrial E3 ubiquitin–protein ligase 1 (MUL1) (Rojansky et al., 2016; Yun et al., 2014), ariadne RBR E3 ubiquitin–protein ligase 1 (ARIH1) (Villa et al., 2017) and membrane-associated ring-CH-type finger 5 (MARCHF5, also known as MITOL) (Koyano et al., 2019).

Ubiquitylated OMM proteins are then recognized by autophagy receptors of the SLR family, which includes sequestosome 1 (SQSTM1, hereafter referred to as p62), NBR1, optineurin (OPTN), Tax1-binding protein 1 (TAX1BP1) and calcium-binding and coiled-coil domain 2 (CALCOCO2, hereafter referred to as NDP52). The SLRs all have specific ubiquitin-binding domains, as well as an LC3-interacting region (LIR) that facilitates their interaction with ATG8 proteins in the autophagosome membrane (Dikic and Elazar, 2018; Johansen and Lamark, 2020) (see Box 2). NDP52 and OPTN have been found to be absolutely required for PINK1/parkin-dependent mitophagy in HeLa cells (Lazarou et al., 2015), but other SLRs have also been implicated in mitophagy – for instance, p62 in macrophages and leukemia cells (Nguyen et al., 2019; Zhong et al., 2016). The interaction of SLRs with an ATG8 protein can be modulated by phosphorylation of residues within and surrounding the core LIR motif (Box 2). As an example, the function of OPTN in parkin-dependent mitophagy is regulated by phosphorylation mediated by both TANK-binding kinase 1 (TBK1) and unc-51 like autophagy activating kinase 1 (ULK1) (Harding et al., 2021). The pro-autophagic autophagy and beclin 1 regulator 1 (AMBRA1) also has an LIR and becomes recruited to the OMM of depolarized mitochondria, where it promotes PINK1 stability and interacts with ATG8s to induce mitophagy (Di Rienzo et al., 2022; Strappazzon et al., 2015). The interaction between AMBRA1 and ATG8 proteins is positively regulated by both ubiquitylation and phosphorylation (Di Rita et al., 2018).

In addition to their role in bridging polyubiquitylated mitochondria to the phagophore membrane via binding to ubiquitin and ATG8s, it has recently been found that SLRs promote *de novo* phagophore membrane biogenesis by recruiting the ULK1 kinase complex (also known as the ULK1/ATG1 kinase complex; Box 1) (Lazarou et al., 2015; Ravenhill et al., 2019; Turco et al., 2019; Vargas et al., 2019). For PINK1/parkin-dependent mitophagy, NDP52 has been found to promote mitochondrial recruitment of FIP200 (also known as RB1-inducible coiled-coil 1, RB1CC1) (Vargas et al., 2019), likely by binding of its LIR domain to the claw domain of FIP200 (Fu et al., 2021), further triggering membrane binding of the ULK1 complex (Shi et al., 2020). Furthermore, binding of the SKICH domain of NDP52 to FIP200 is essential for xenophagy (Ravenhill et al., 2019), suggesting that this is also important for mitophagosome biogenesis.

Binding of autophagy receptors to ubiquitylated OMM proteins can be further modulated by their interaction with internal mitochondrial proteins and lipids that function as specific ‘eat me’ signals on the surface of damaged mitochondria. Mitochondrial recruitment of NDP52, as well as other SLRs, has been found to depend on binding to the mitochondrial matrix proteins NIPSNAP1 and NIPSNAP2, which become stabilized on the surface of depolarized mitochondria (Princely Abudu et al., 2019) (see poster). Moreover, the IMM protein PHB2 interacts with LC3 upon proteasomal-dependent OMM rupture (Wei et al., 2017) and has been implicated in stabilization of PINK1 on the OMM following membrane depolarization (Yan et al., 2020).

It is interesting to note that ATG8s are important, but not essential, for cargo selection during PINK1/parkin-dependent mitophagy (Nguyen et al., 2016; Vaites et al., 2018). In line with this, LIR-independent recruitment of SLRs to mitochondrial phagophores appears to amplify the mitophagy signal, as the SLRs promote further recruitment of the cargo receptors and autophagy machineries (Padman et al., 2019). Thus, complex multivalent interactions of SLRs and ATG8s with mitochondrial proteins and lipids underlie the coordination of events required for mitophagosome membrane biogenesis around damaged or dysfunctional mitochondria. Little is known about how SLR-dependent mitophagy is turned off, but several deubiquitylating enzymes (DUBs) have been shown to antagonize parkin-mediated ubiquitylation and mitophagy, including the ubiquitin-specific peptidases USP15, USP30, USP33, USP35 and USP36 (Bingol et al., 2014; Cornelissen et al., 2014; Geisler et al., 2019; Gersch et al., 2017; Niu et al., 2020; Wang et al., 2015). Furthermore, protein phosphatase with EF-hand domain 2 (PPEF2), which dephosphorylates ubiquitin at Ser65, has been shown to inhibit PINK1-dependent mitophagy (Wall et al., 2019).

#### PINK1/parkin-independent SLR mitophagy

Several E3 ubiquitin ligases appear to have a redundant function with parkin to promote SLR-dependent mitophagy, including TNF receptor-associated factor 2 (TRAF2) (Yang et al., 2015), CIAP (also known as BIRC2) (Zachari et al., 2019) and the α isoform of tripartite motif-containing protein 5 (TRIM5α) (Saha et al., 2022). Induction of mitophagy by the lactone ivermectin, causing a rapid fragmentation of mitochondria, results in mitochondrial recruitment of OPTN. Mitochondrial OPTN recruitment requires TBK1-mediated activation of FIP200 and ATG13, but not the activity of the kinases ULK1 and ULK2 (Zachari et al., 2019). At mitochondria, OPTN further promotes *de novo* synthesis of mitophagosomes by recruiting vesicles containing ATG9A (Yamano et al., 2020), a lipid scramblase required for phagophore biogenesis (Matoba et al., 2020).

### SLR-independent mitophagy – a mitochondrial receptor pathway

Induction of mitophagy in response to other cellular or environmental stressors, such as iron depletion and hypoxia, does not appear to depend on ubiquitylation of mitochondrial OMM proteins and SLRs. However, there seems to be a general requirement for integral mitochondrial proteins that have a LIR domain that can interact directly with ATG8 proteins in the phagophore membrane. Such mitophagy receptors are generally mitochondrial proteins anchored on the cytosolic face of the OMM and include BNIP3 (Quinsay et al., 2010), BNIP3L (Novak et al., 2010; Sandoval et al., 2008; Schwarten et al., 2009), FUNDC1 (Chen et al., 2016; Liu et al., 2012), BCL2-like 13 (BCL2L13) (Murakawa et al., 2015), FKBP prolyl isomerase 8 (FKBP8) (Bhujabal et al., 2017) and NLR family member X1 (NLRX1) (Zhang et al., 2019) in humans (see poster). In yeast, the OMM protein Atg32 interacts with Atg8 and Atg11 to mediate mitophagy (Kanki et al., 2009; Okamoto et al., 2009).

Although our knowledge about how the mitophagy functions of these receptors are regulated is still sparse, it is becoming evident that their activity is tightly regulated at several levels. BNIP3L and BNIP3 levels are transcriptionally upregulated by HIF1α during hypoxia (Bellot et al., 2009) and iron depletion (Allen et al., 2013; Zhao et al., 2020). At basal levels, expression of both BNIP3L and FUNDC1 are regulated by miR137, a microRNA that is downregulated in response to hypoxia, thereby preventing mitophagy under normoxia conditions (Li et al., 2014). As is the case for SLRs, the affinity of mitochondrial mitophagy receptors towards ATG8 proteins is closely controlled by phosphorylation of their LIR motifs (Poole et al., 2021; Rogov et al., 2017; Zhu et al., 2013). As an example, the LIR motif of BNIP3 is enclosed by two serine residues (Ser17 and Ser24), where phosphorylation of Ser17 is essential for BNIP3 binding to LC3B, whereas phosphorylation of both Ser17 and Ser24 facilitates its binding to GABARAPL2, further promoting mitophagy (see Box 2) (Zhu et al., 2013). Intriguingly, the interaction of FUNDC1 with ATG8 proteins is negatively regulated under normoxic conditions by phosphorylation of the LIR-surrounding residues Ser13 and Tyr18 [by casein kinase 2 (CK2) and SRC kinase, respectively]. These residues become dephosphorylated upon hypoxia, when ULK1-mediated phosphorylation of Ser17 promotes the interaction of FUNDC1 with LC3 and induction of mitophagy (Chen et al., 2014; Liu et al., 2012; Wu et al., 2014) (see poster). It is interesting to note that phosphorylation of FUNDC1 on Tyr18 decreases its interaction with NIPSNAP1 and NIPSNAP2 on the OMM of damaged mitochondria upon ischemic reperfusion injury, resulting in an accumulation of damaged mitochondria (Li et al., 2021). Thus, the phosphorylation status of FUNDC1 appears to regulate mitophagy in both a positive and negative manner.

Although mitochondrial mitophagy receptors can be phosphorylated by the ULK1 kinase, it remains unknown whether they interact directly with FIP200, as is seen for SLRs (Ravenhill et al., 2019; Turco et al., 2019; Vargas et al., 2019), to facilitate mitochondrial recruitment of the ULK1 complex and *de novo* mitophagosome formation. The mitochondria-localized protein kinase C δ (PRKCD) has recently been shown to stimulate mitochondrial recruitment of ULK1 and ATG13 upon hypoxia-induced mitophagy, but its specific substrates remain unknown (Munson et al., 2021).

Dimerization of the BNIP3L receptor further promotes recruitment of the autophagy machinery and mitophagy progression (Marinkovic et al., 2021), suggesting that receptor oligomerization might increase the ATG8 binding avidity. In support of this, the intracellular bacterial pathogen *Listeria monocytogenes* has been found to induce mitophagy in macrophages through the virulence factor listeriolysin O (LLO), which promotes oligomerization of NLRX1 and binding of its LIR motif to LC3, thereby aiding bacterial survival by induction of mitophagy and reducing ROS levels (Zhang et al., 2019).

The phosphorylation status of mitophagy receptors can also modulate their interaction with proteins that regulate mitochondrial morphology. For instance, phosphorylation of FUNDC1 on Ser13 promotes its interaction with the mitochondrial dynamin-like GTPase OPA1 and decreases its interaction with the mitochondrial dynamin-like GTPase DRP1 (also known as DNM1L), thus inhibiting mitochondrial fission (Chen et al., 2016). In contrast, the mitochondrial serine/threonine protein phosphatase PGAM family member 5 (PGAM5) dephosphorylates FUNDC1 Ser13 and DRP1, thereby stimulating mitochondrial fission (Chen et al., 2014; Sugo et al., 2018). The correlation between mitochondrial morphology and mitophagy is, however, not so clear. Several studies have found that mitochondrial fission is important for mitophagy (Abeliovich et al., 2013; Ikeda et al., 2015; Kageyama et al., 2014; Mao et al., 2013; Rambold et al., 2011; Tanaka et al., 2010; Twig et al., 2008), but other studies have reported that mitophagosome formation occurs independently of mitochondrial fission (Burman et al., 2017; Yamashita et al., 2016). This is likely dependent on the type of mitophagy-inducing signal, as well as cell or tissue type. Moreover, in addition to the mitochondrial fission and fusion machinery, it is possible that the endosomal sorting complexes required for transport (ESCRT) machinery, which is involved in mitophagosome closure (Zhen et al., 2020), can facilitate ‘pinching off’ of small fragments of mitochondria. Additionally, sequestration of small pieces of mitochondria through ‘piecemeal mitophagy’ (see below) or budding of mitochondria-derived vesicles (MDVs) contribute to the turnover of specific mitochondrial fragments that can be targeted for lysosomal degradation.

In addition to protein-based mitophagy receptors, lipids might also play a similar critical role. For example, the IMM lipid cardiolipin translocates to the OMM following mitochondrial injury, and once on the surface it can interact with LC3 to facilitate mitophagy (Chu et al., 2013).

### Piecemeal mitophagy

While macromitophagy is believed to involve sequestration and lysosomal targeting of whole mitochondria, piecemeal mitophagy involves the sequestration and delivery of selected mitochondrial cargo to lysosomes, seemingly in a DRP1-independent manner (Abudu et al., 2021; Le Guerroué et al., 2017). Mechanistically, little is known about this type of mitophagy, but it appears to rely on SLRs and ATG8 proteins (see poster). The OMM protein metaxin 1 (MTX1) has been found to directly interact with LC3C and p62 to promote phagophore sequestration of mitochondrial fragments (Le Guerroué et al., 2017). Moreover, the sorting and assembly machinery (SAM) component SAMM50 has been found to function as a receptor for basal mitophagy of components of the SAM and mitochondrial contact site and cristae organizing system (MICOS) complexes through its interaction with p62 and ATG8 proteins (Abudu et al., 2021; Le Guerroué et al., 2017).

### Alternative mitolysosomal pathways

#### ATG8 conjugation-independent mitophagy

The autophagy ubiquitin-like conjugation system is essential for conventional macroautophagy (see Box 1). However, instances of mitophagy have been observed in cells where this system has been inhibited through loss of ATG5 or ATG7. Certain cytotoxic stressors, such as etoposide, in ATG5-null mouse embryonic fibroblasts have been found to induce a form of macroautophagy that appears to involve autophagosomes derived from Golgi membranes and requires the small GTPase RAB9A (Nishida et al., 2009). Although much less is known about the mechanism, this process does require some of the conventional autophagy machinery, including the ULK1 complex and class III phosphatidylinositol 3-kinase complex I (also known as the VPS34 complex or PIK3C3–C1 complex; Box 1). Of note, this pathway appears to be relevant for certain physiological instances of mitophagy and can occur during reticulocyte development (Honda et al., 2014; Nishida et al., 2009) and ischemic stress in the heart (Saito et al., 2019).

#### Mitochondria-derived vesicles

Mitochondrial components can also be delivered to lysosomes via a conventional membrane trafficking pathway, where MDVs bud from mitochondria and fuse with the endolysosomal system (Soubannier et al., 2012; Sugiura et al., 2014) (see poster). Although this is a separate pathway and is independent of the core autophagy machinery, it has a close relationship to mitophagy. It can be stimulated under conditions that also promote mitophagy, such as mitochondrial depolarization and oxidative stress (Soubannier et al., 2012), and may even require some of the same machinery, such as PINK1 and parkin (McLelland et al., 2014). The MDV pathway can also be upregulated under conditions where conventional mitophagy is blocked, where it is thought to act in a compensatory manner (Towers et al., 2021).

## Concluding remarks and future perspectives

Mammalian mitophagy is clearly a physiologically important process with an *in vivo* prevalence that has been highlighted by novel reporter models allowing high-resolution visualization of mitophagy within specialized cells of distinct tissues (McWilliams et al., 2016; Sun et al., 2015). However, despite uncovering these physiological instances, less is known about which mitophagy pathway is responsible. Likewise, although we have advanced our understanding of the mechanisms involved in lysosomal degradation of mitochondrial components, there are still many open questions. Is the mitochondrial cargo the same for different types of mitophagy? How can we distinguish different mitophagy pathways mechanistically, especially if distinct pathways can occur at the same time? It is important to keep in mind that key players of mitophagy might be regulated oppositely under different metabolic conditions – for example, phosphorylation of FUNDC1 under normoxic versus hypoxic conditions – thus making it difficult to draw conclusions from protein-depletion experiments. Moreover, as mitochondrial turnover is often studied in the context of severe damage to mitochondria in cell culture, it will be important to characterize the different pathways under more physiologically relevant conditions, as well as how they are regulated in time (during aging or disease onset) and space (in different cells and tissues). Such knowledge is essential to eventually be able to target mitophagy therapeutically and to address whether lysosomal degradation of surplus and/or dysfunctional mitochondria indeed protects against disease development.

## Panel 1. Diversity of mitophagy pathways

Panel 1. Diversity of mitophagy pathways

## Panel 2. HIF1 regulation

Panel 2. HIF1 regulation

## Panel 3. General mechanism of autophagosome formation and growth

Panel 3. General mechanism of autophagosome formation and growth

## Panel 4. Summary of mitophagy receptor proteins

Panel 4. Summary of mitophagy receptor proteins

## Panel 5. SLR-independent mitophagy

Panel 5. SLR-independent mitophagy

## Panel 6. PINK1/parkin-dependent SLR mitophagy

Panel 6. PINK1/parkin-dependent SLR mitophagy

## Panel 7. PINK1 stabilization upon depolarization

Panel 7. PINK1 stabilization upon depolarization

## Panel 8. PINK1/parkin-independent SLR mitophagy

Panel 8. PINK1/parkin-independent SLR mitophagy

## Panel 9. Piecemeal mitophagy

Panel 9. Piecemeal mitophagy

## Panel 10. Alternative mitolysosomal pathways

Panel 10. Alternative mitolysosomal pathways

## Panel 11. Mitochondrial dynamics

Panel 11. Mitochondrial dynamics

## Panel 12. Additional roles of ubiquitylation

Panel 12. Additional roles of ubiquitylation
