# Supplementary figures and images for: Diversity of mitophagy pathways at a glance
Source: J Cell Sci. 2022 Dec 12;135(23):jcs259748. doi: 10.1242/jcs.259748 (PMC10656428; doi:10.1242/jcs.259748)

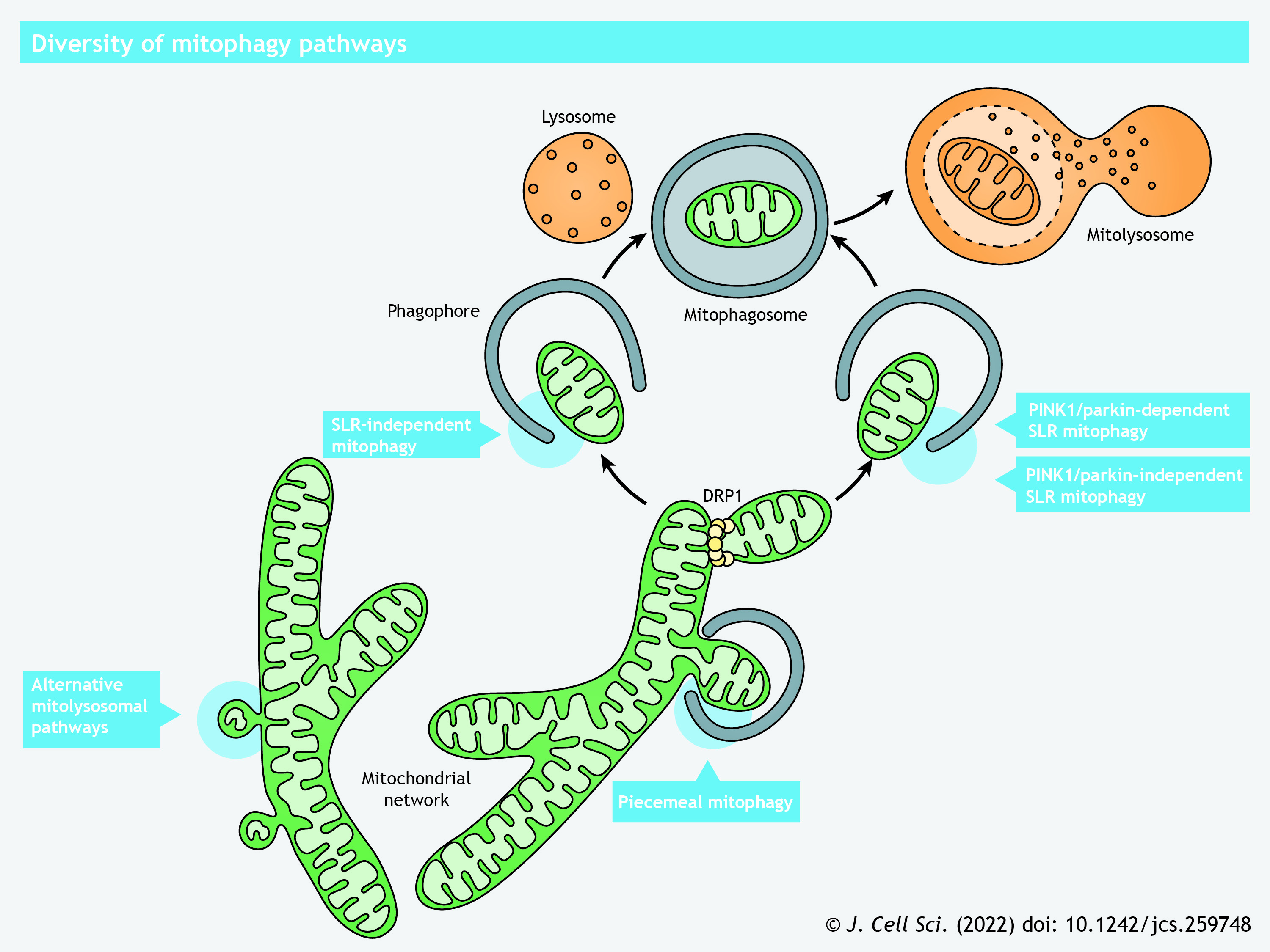

Supplement: Panel 1. Diversity of mitophagy pathways [file joces-135-259748-s1.jpg]

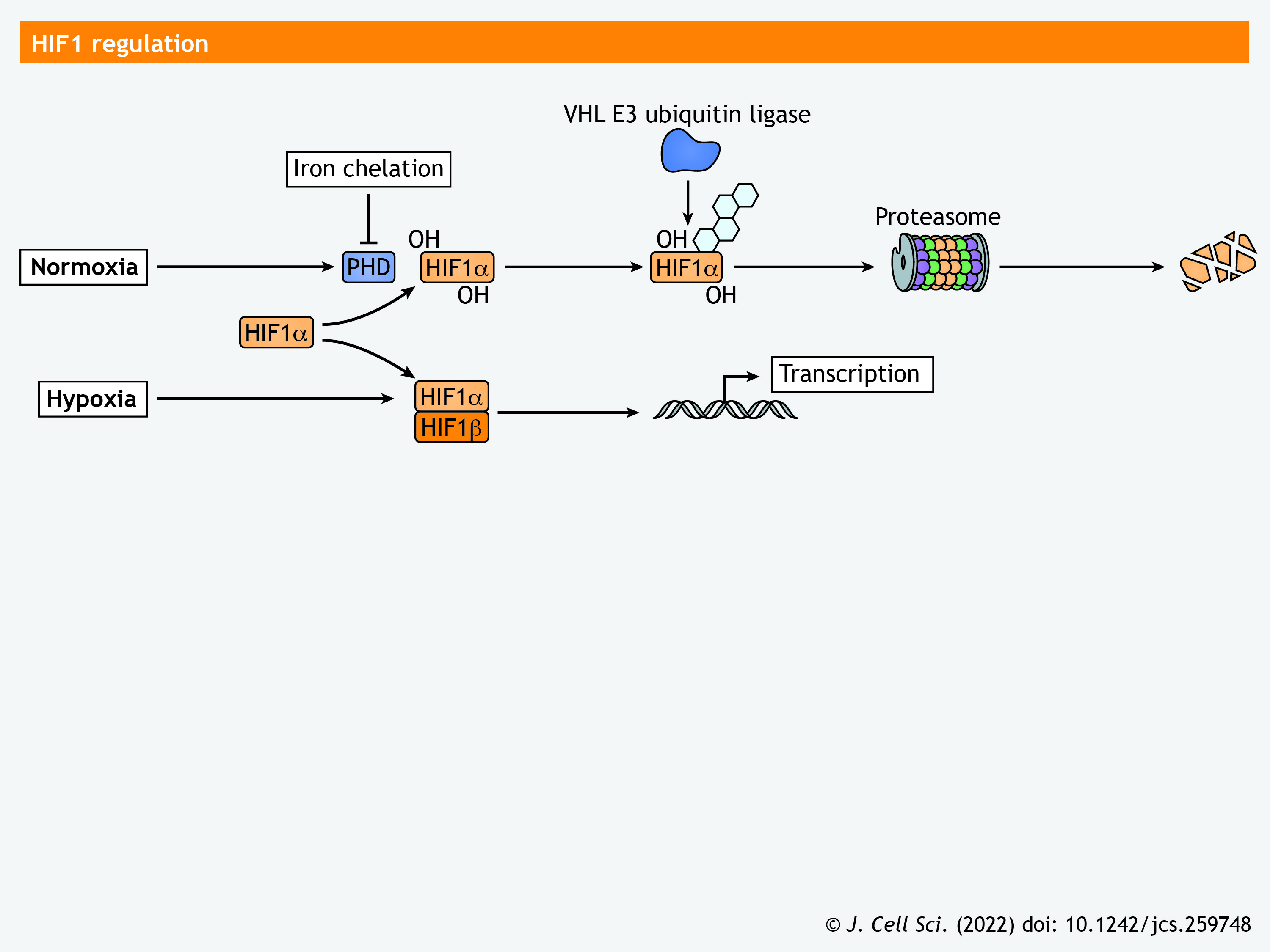

Supplement: Panel 2. HIF1 regulation [file joces-135-259748-s2.jpg]

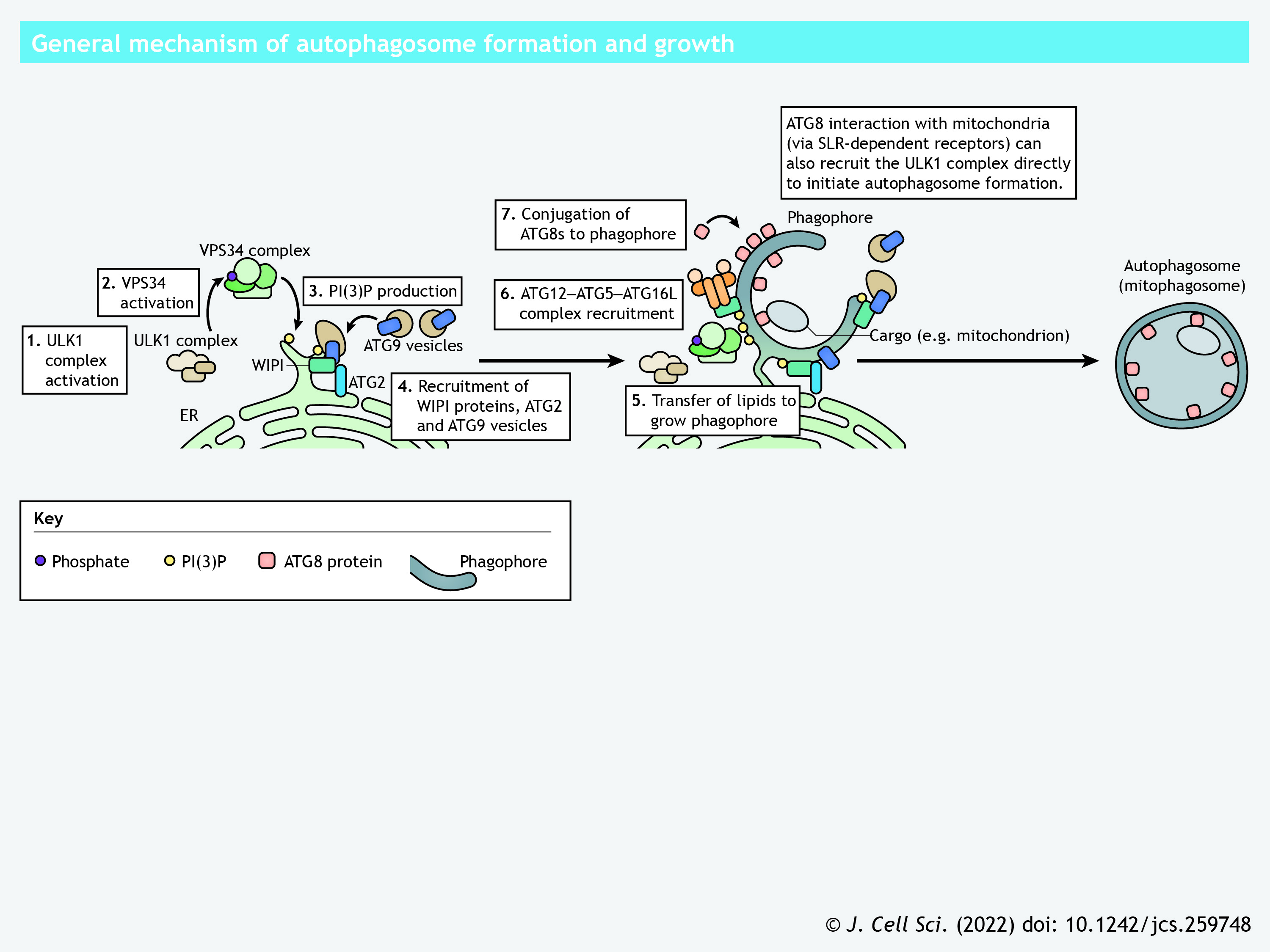

Supplement: Panel 3. General mechanism of autophagosome formation and growth [file joces-135-259748-s3.jpg]

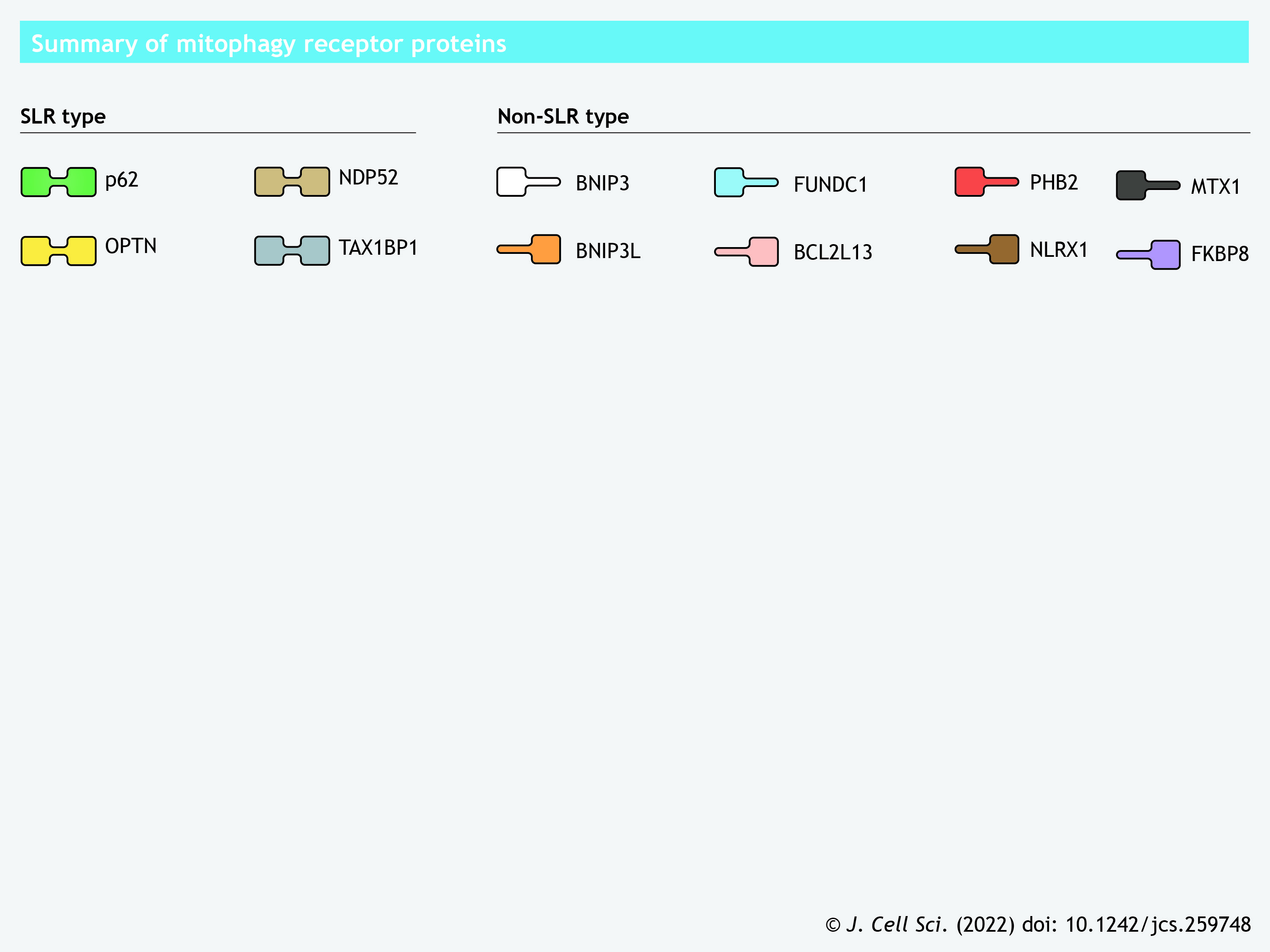

Supplement: Panel 4. Summary of mitophagy receptor proteins [file joces-135-259748-s4.jpg]

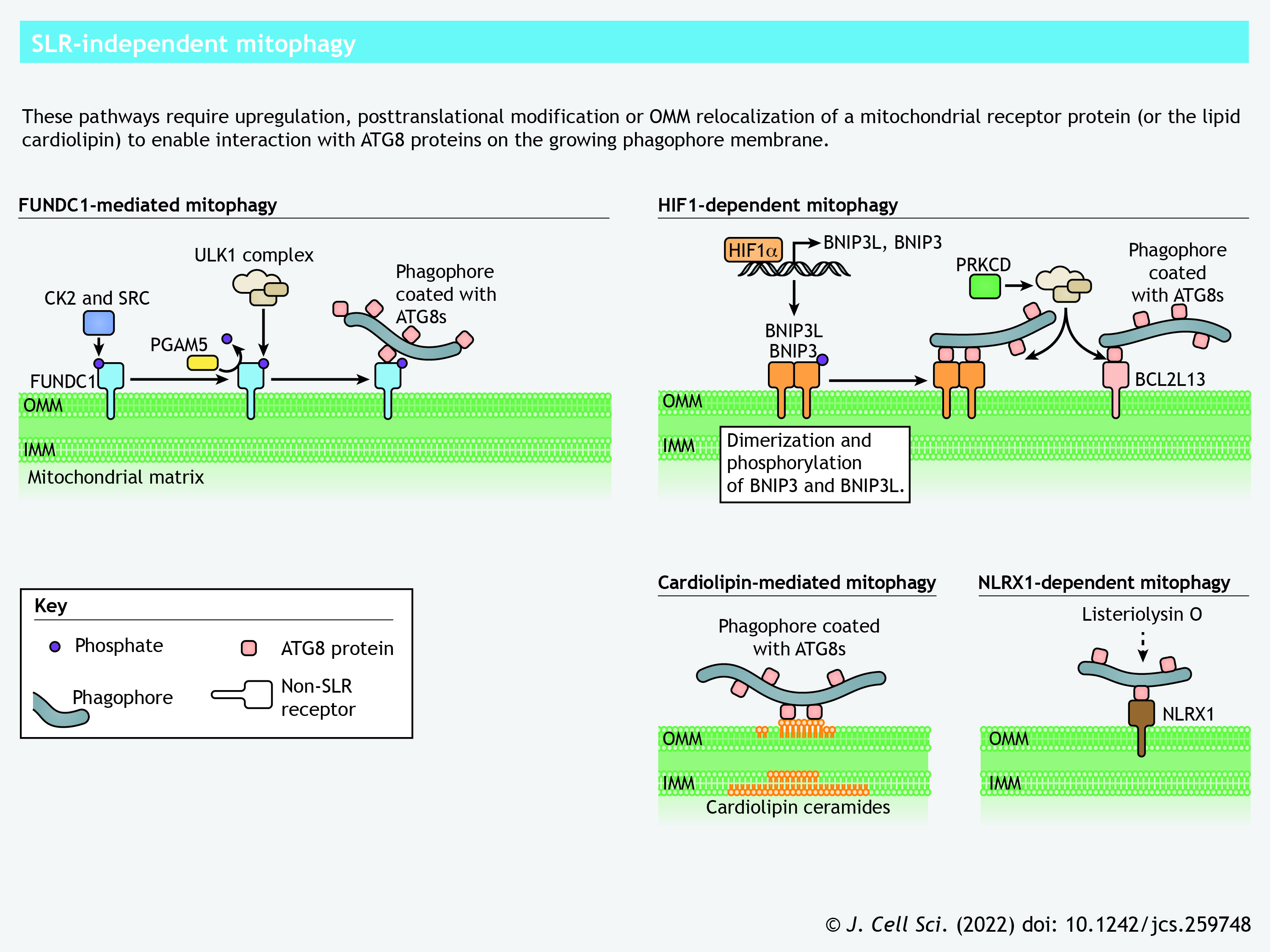

Supplement: Panel 5. SLR-independent mitophagy [file joces-135-259748-s5.jpg]

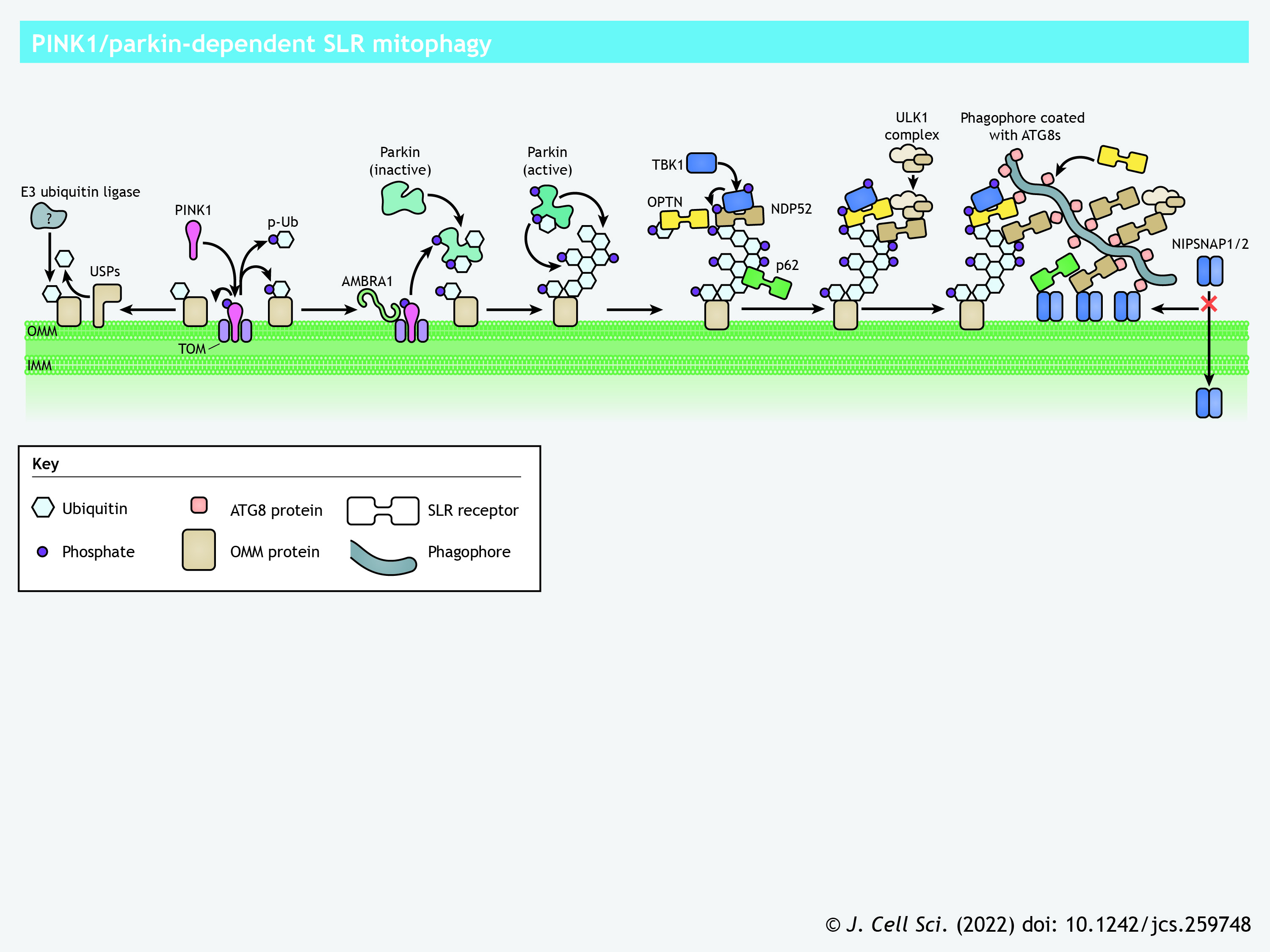

Supplement: Panel 6. PINK1/parkin-dependent SLR mitophagy [file joces-135-259748-s6.jpg]

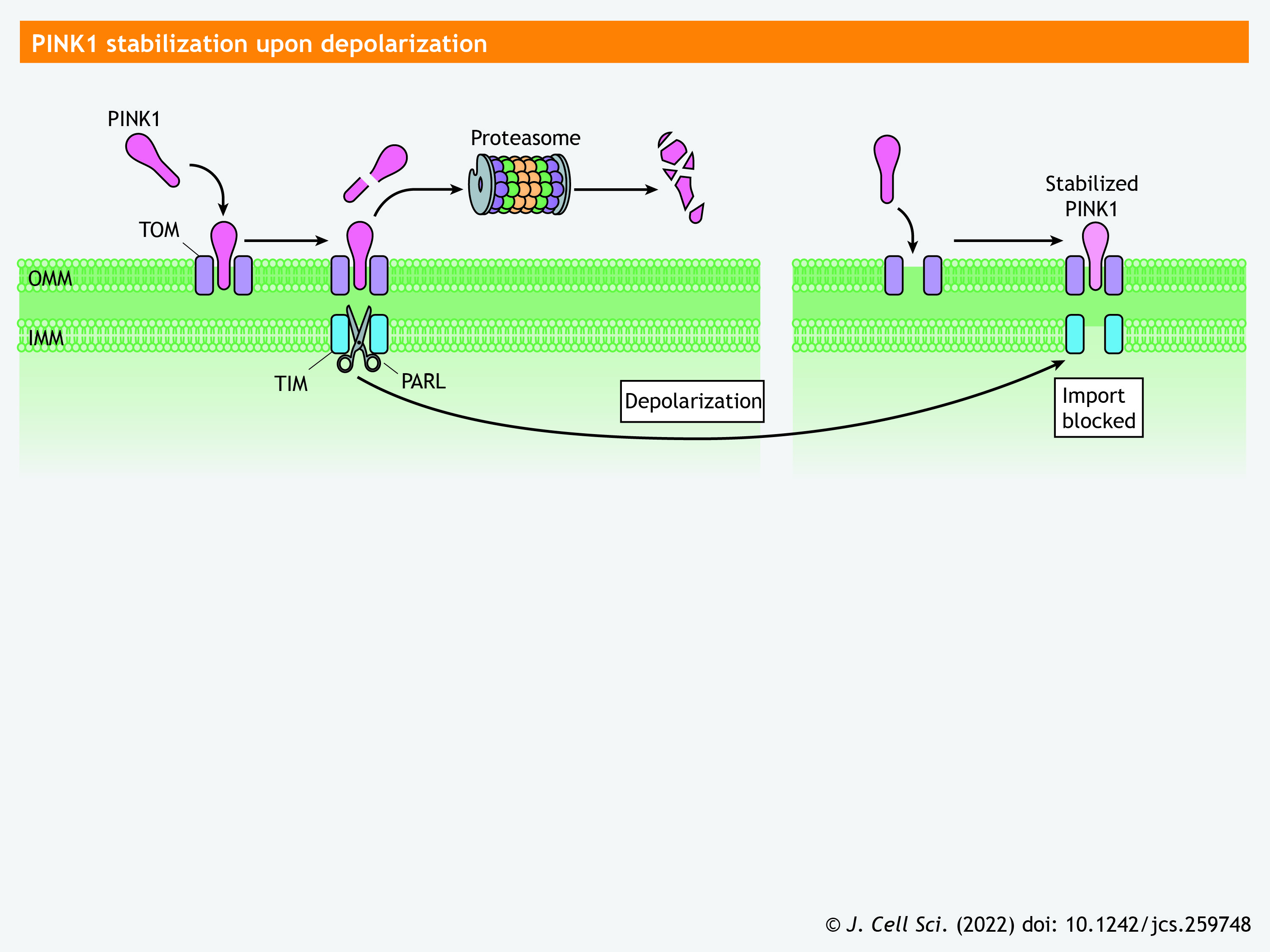

Supplement: Panel 7. PINK1 stabilization upon depolarization [file joces-135-259748-s7.jpg]

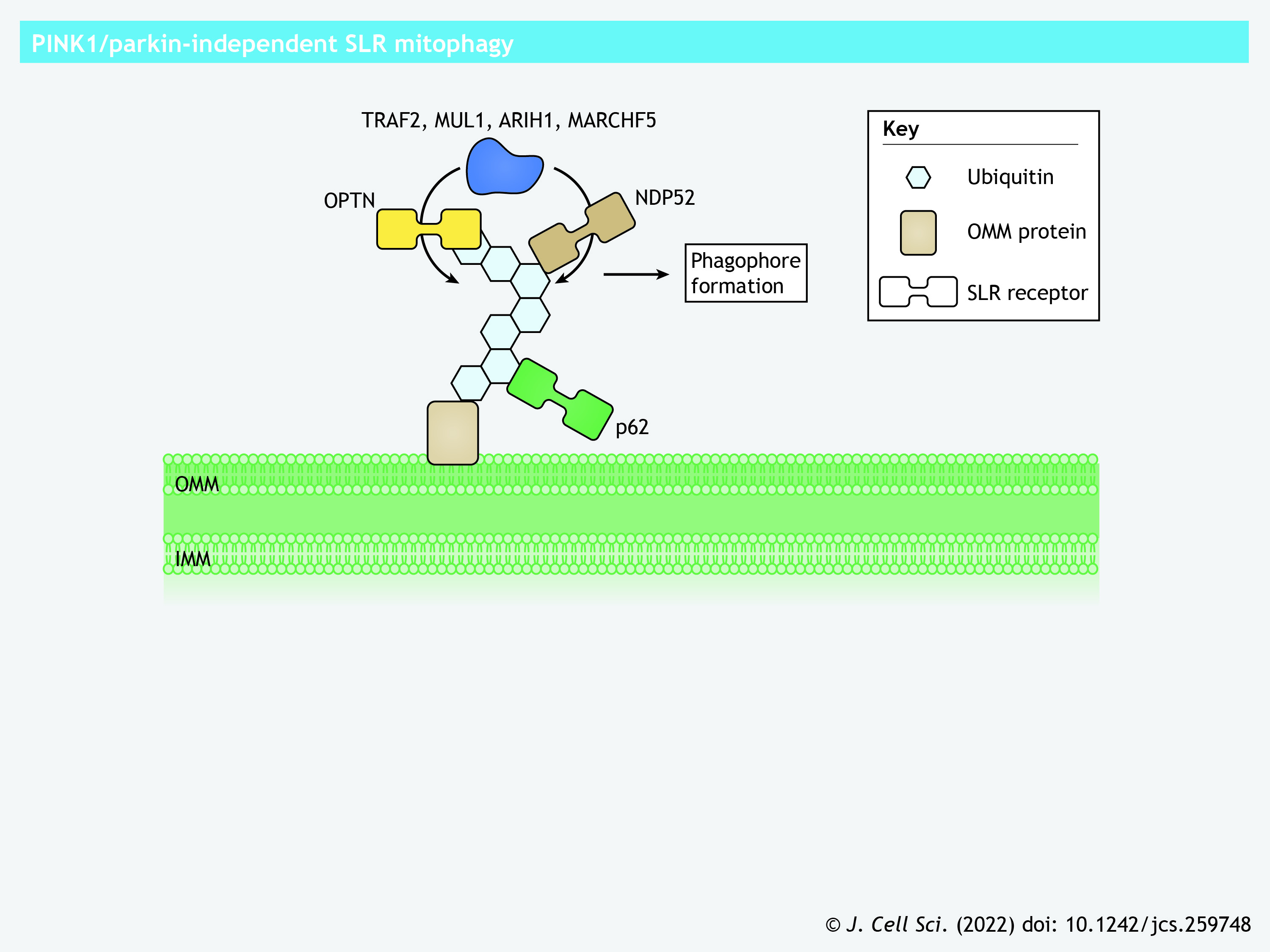

Supplement: Panel 8. PINK1/parkin-independent SLR mitophagy [file joces-135-259748-s8.jpg]

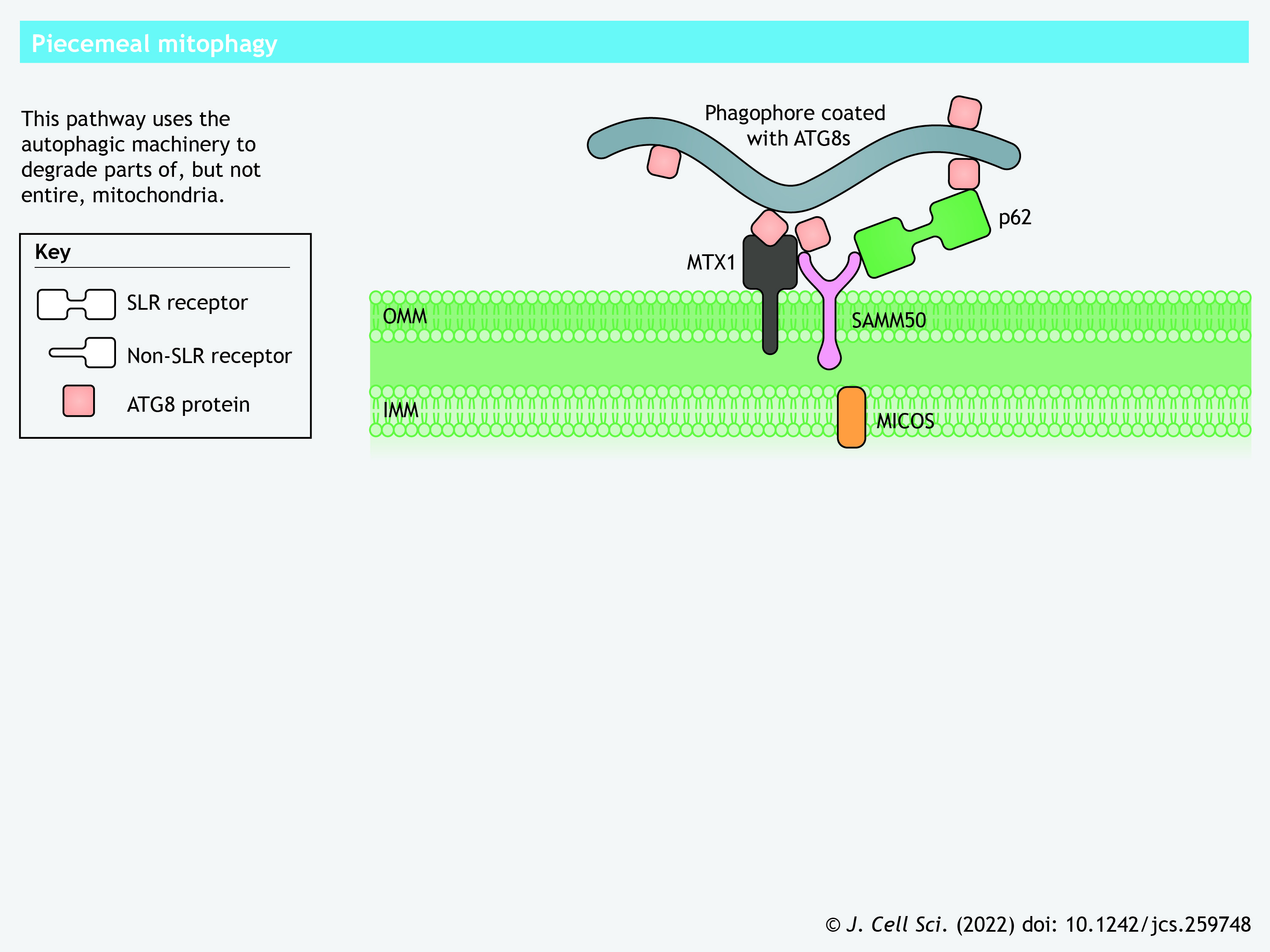

Supplement: Panel 9. Piecemeal mitophagy [file joces-135-259748-s9.jpg]

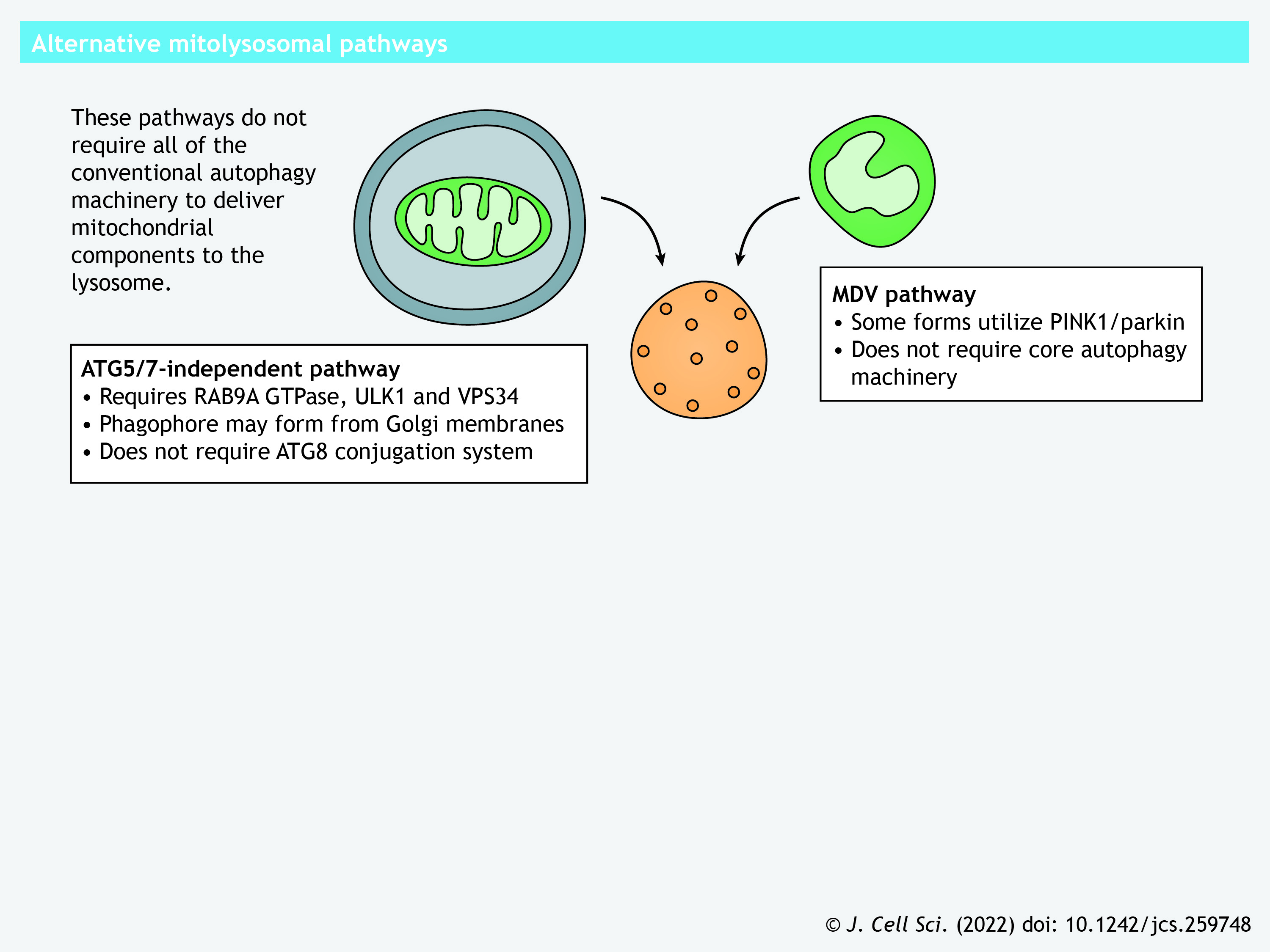

Supplement: Panel 10. Alternative mitolysosomal pathways [file joces-135-259748-s10.jpg]

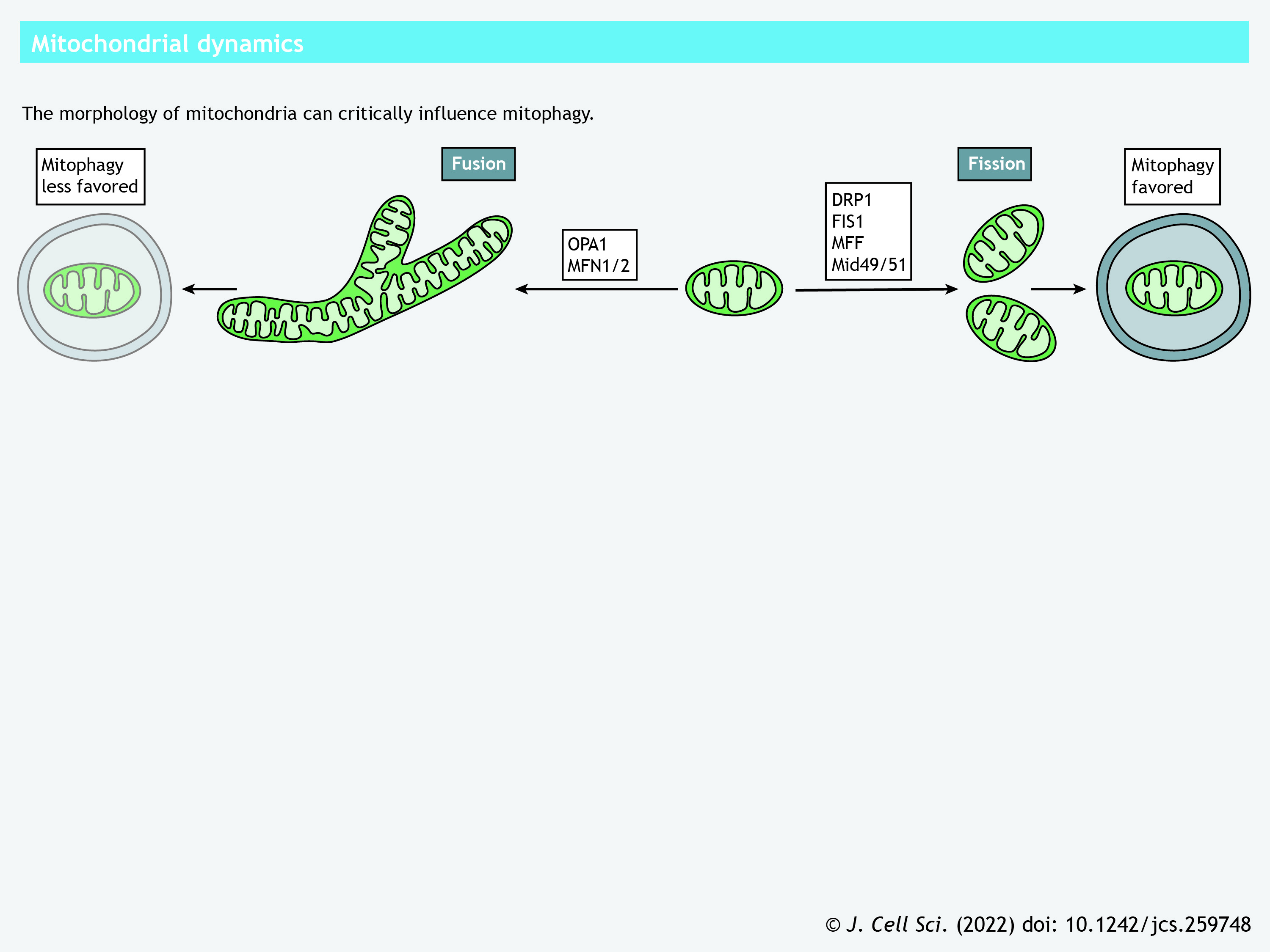

Supplement: Panel 11. Mitochondrial dynamics [file joces-135-259748-s11.jpg]

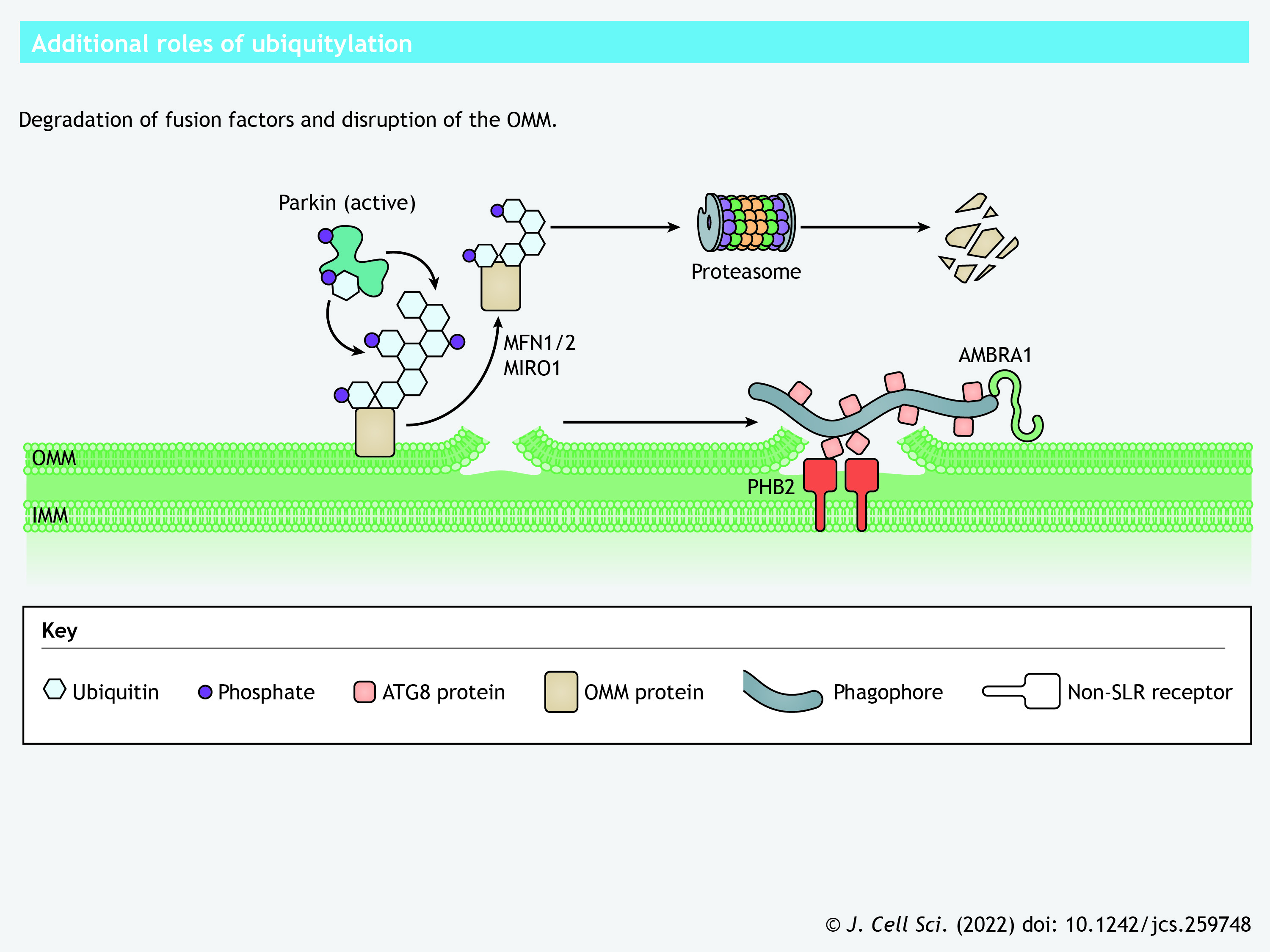

Supplement: Panel 12. Additional roles of ubiquitylation [file joces-135-259748-s12.jpg]
